# Supplementary material for: Modeling glioblastoma heterogeneity as a dynamic network of cell states
Source: Mol Syst Biol. 2021 Sep 16;17(9):e10105. doi: 10.15252/msb.202010105 (PMC8444284; doi:10.15252/msb.202010105)
Supplement: Supplementary file 6 — Source Data for Figure 5 [file MSB-17-e10105-s004.zip › Figure5A_sourcedata/GSEA_3071/nosigdb_stateIV.GseaPreranked.1622795164626/RICHARDS_NEFTEL_CELL_2019_G1_S.html]

Details for gene set RICHARDS\_NEFTEL\_CELL\_2019\_G1\_S[GSEA]

|  || Dataset | state53071 |
| Phenotype | NoPhenotypeAvailable |
| Upregulated in class | na\_neg |
| GeneSet | RICHARDS\_NEFTEL\_CELL\_2019\_G1\_S |
| Enrichment Score (ES) | -0.78878796 |
| Normalized Enrichment Score (NES) | -3.0453238 |
| Nominal p-value | 0.0 |
| FDR q-value | 0.0 |
| FWER p-Value | 0.0 |
Table: GSEA Results Summary

  

Fig 1: Enrichment plot: RICHARDS\_NEFTEL\_CELL\_2019\_G1\_S      
 Profile of the Running ES Score & Positions of GeneSet Members on the Rank Ordered List

  

| PROBE | GENE SYMBOL | GENE\_TITLE | RANK IN GENE LIST | RANK METRIC SCORE | RUNNING ES | CORE ENRICHMENT || 1 | RNASEH2A |  |  | 2595 | -0.282 | -0.4824 | No |
| 2 | MCM2 |  |  | 3240 | -0.366 | -0.5911 | No |
| 3 | PKMYT1 |  |  | 3311 | -0.377 | -0.5903 | No |
| 4 | TMEM106C |  |  | 3669 | -0.438 | -0.6418 | No |
| 5 | ASF1B |  |  | 4328 | -0.606 | -0.7441 | No |
| 6 | CCNE2 |  |  | 4564 | -0.714 | -0.7621 | Yes |
| 7 | DUT |  |  | 4642 | -0.762 | -0.7483 | Yes |
| 8 | CDC45 |  |  | 4702 | -0.798 | -0.7297 | Yes |
| 9 | MCM7 |  |  | 4704 | -0.800 | -0.7000 | Yes |
| 10 | MELK |  |  | 4758 | -0.835 | -0.6789 | Yes |
| 11 | MCM3 |  |  | 4834 | -0.901 | -0.6595 | Yes |
| 12 | GMNN |  |  | 4873 | -0.939 | -0.6316 | Yes |
| 13 | CENPM |  |  | 4881 | -0.947 | -0.5976 | Yes |
| 14 | GINS2 |  |  | 4897 | -0.962 | -0.5645 | Yes |
| 15 | MCM4 |  |  | 4947 | -1.011 | -0.5360 | Yes |
| 16 | RRM1 |  |  | 4990 | -1.074 | -0.5039 | Yes |
| 17 | FEN1 |  |  | 5014 | -1.113 | -0.4667 | Yes |
| 18 | AURKB |  |  | 5016 | -1.118 | -0.4251 | Yes |
| 19 | TK1 |  |  | 5020 | -1.126 | -0.3837 | Yes |
| 20 | CDCA5 |  |  | 5023 | -1.133 | -0.3417 | Yes |
| 21 | PCNA |  |  | 5060 | -1.214 | -0.3032 | Yes |
| 22 | CENPK |  |  | 5091 | -1.267 | -0.2616 | Yes |
| 23 | ZWINT |  |  | 5115 | -1.321 | -0.2166 | Yes |
| 24 | CKS1B |  |  | 5121 | -1.338 | -0.1676 | Yes |
| 25 | RAD51AP1 |  |  | 5133 | -1.375 | -0.1183 | Yes |
| 26 | RRM2 |  |  | 5232 | -1.875 | -0.0669 | Yes |
| 27 | HIST1H4C |  |  | 5249 | -2.081 | 0.0078 | Yes |
Table: GSEA details [plain text format]

  

Fig 2: RICHARDS\_NEFTEL\_CELL\_2019\_G1\_S: Random ES distribution      
 Gene set null distribution of ES for **RICHARDS\_NEFTEL\_CELL\_2019\_G1\_S**
